# Supplementary figures and images for: Aggregation of CAT tails blocks their degradation and causes proteotoxicity in S. cerevisiae
Source: PLoS One. 2020 Jan 16;15(1):e0227841. doi: 10.1371/journal.pone.0227841 (PMC6964901; doi:10.1371/journal.pone.0227841)

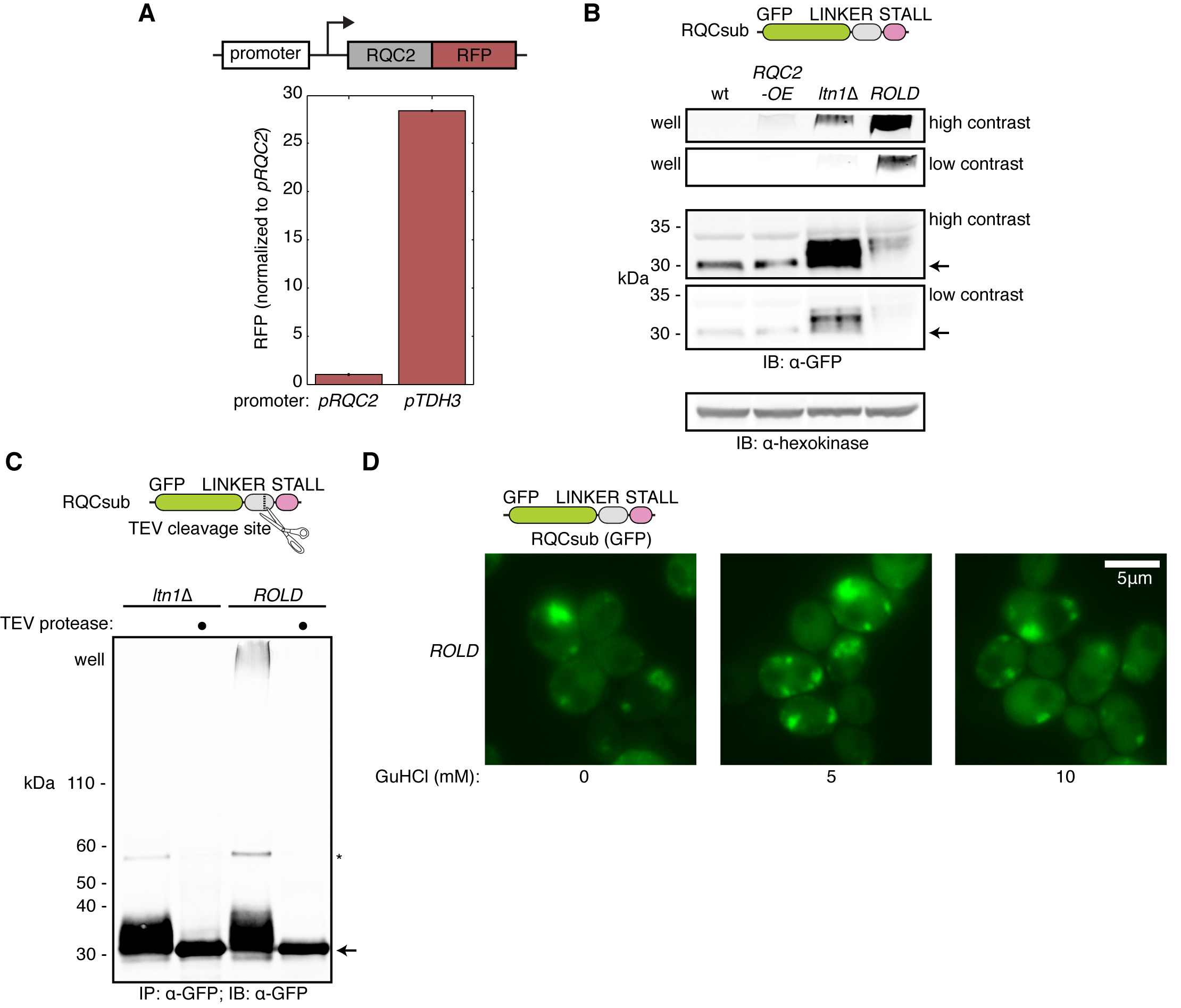

Supplement: S1 Fig — (A) Flow cytometry of cells expressing Rqc2-RFP to assess the degree of Rqc2 overproduction by the TDH3 promoter. Error bars represent s.e.m. from three independent cultures. (B) Lysate IB of RQCsub expressed in ltn1Δ compared to ROLD. (C) Immunoblot of RQCsub immunoprecipitated from ltn1Δ and ROLD lysates with and without tobacco etch virus (TEV) protease treatment. Arrow denotes molecular weight of non-CATylated RQCsub. Asterisk indicates the full-length RQCsub protein product, produced when ribosomes translate through the stall sequence (region past the stall not pictured in schematic). (D) Microscopy of RQCsub expressed in ROLD grown in various concentrations of guanidinium hydrochloride (GuHCl). (TIF) [file pone.0227841.s001.tif]

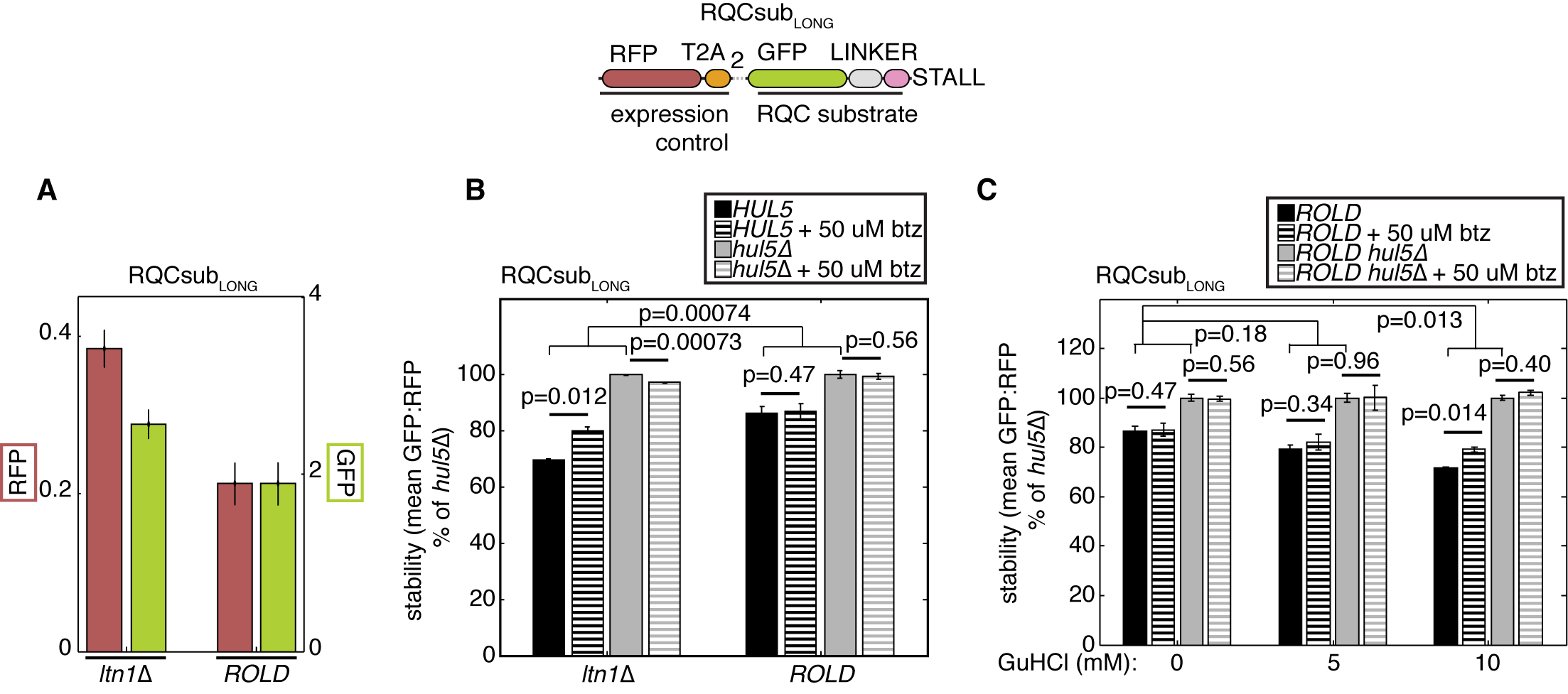

Supplement: S2 Fig — (A) Flow cytometry of cells expressing RQCsubLONG. Error bars indicate s.e.m. from three independent cultures. (B) and (C) Additional data to support Fig 1F–1G. Stability measurements of RQCsubLONG expressed in ROLD cells grown in indicated GuHCl concentrations with additional bortezomib treatment to inhibit the proteasome and HUL5 deletion to measure CAT tail degron activity. Error bars as in A. P-values are indicated above bars. Thick lines indicate paired t-tests, probing the significance of bortezomib (btz)-induced stabilization. Thin lines denote t-tests for particular contrast, measuring how significantly different HUL5 deletion-induced stabilization is under different conditions. (TIF) [file pone.0227841.s002.tif]

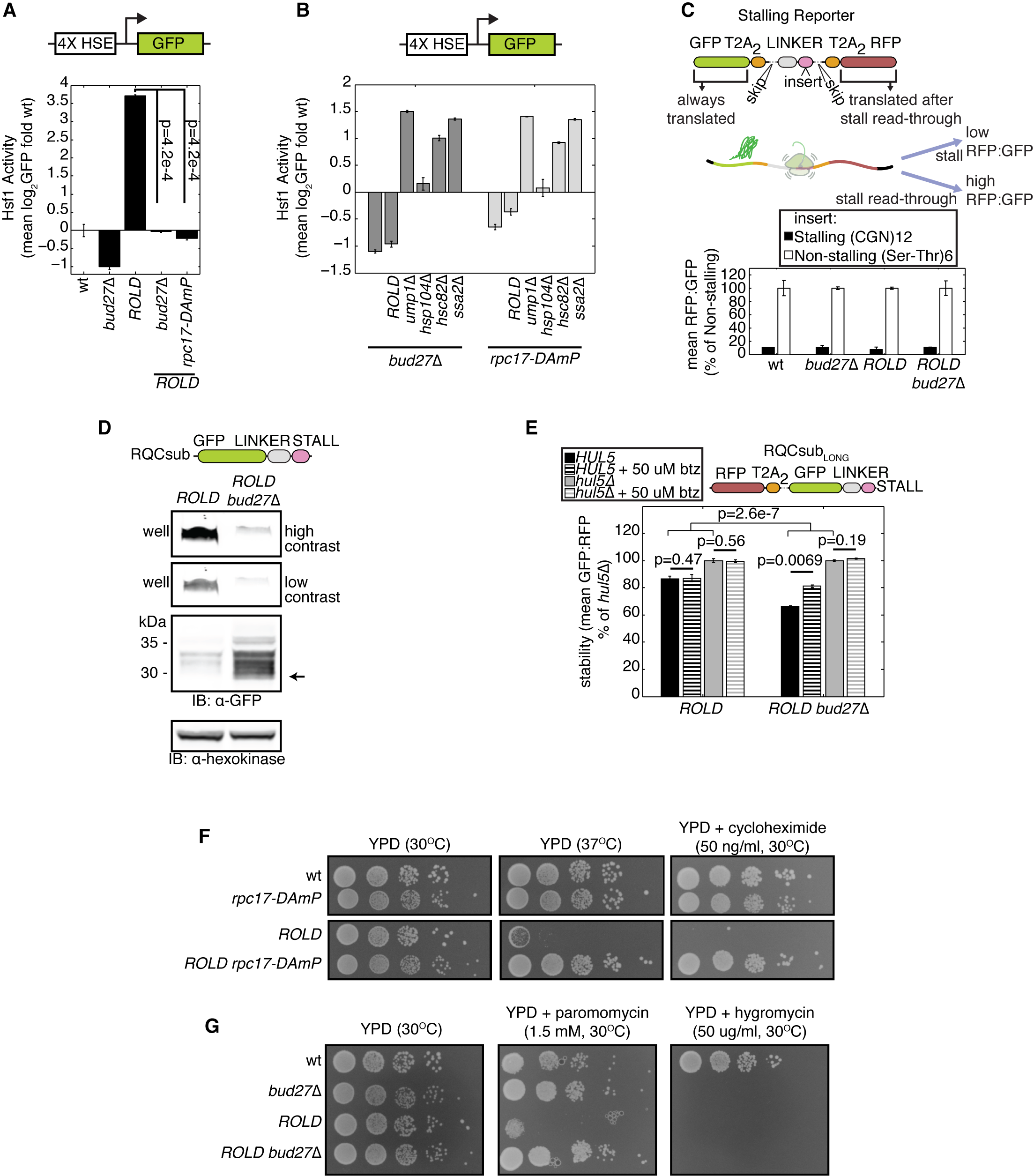

Supplement: S3 Fig — (A) Flow cytometry of cells containing an integrated reporter for Hsf1 activation. Error bars indicate s.e.m. from three independent cultures. P-values from paired t-tests indicated above bars. (B) Flow cytometry of Pol III-perturbed cells containing an integrated reporter for Hsf1 activation. These data are also contained in Fig 3A, but are reordered here to simplify comparisons within two Pol III-perturbed genetic backgrounds. Error bars as in A. (C) Above, schematic of stalling reporter with the same (CGN)12 stalling sequence contained in RQCsub or a non-stalling (Ser-Thr)6 sequence, similar to a reporter used in refs 11 and 12. Below, flow cytometry of stalling and non-stalling reporters expressed in indicated strains. Error bars as in A. (D) IB of lysates containing RQCsub derived from ROLD compared to ROLD bud27Δ. (E) Additional data to support Fig 3E. Stability measurements of RQCsubLONG expressed in indicated strains with bortezomib (btz) treatment to inhibit the proteasome and HUL5 deletion to block CAT tail degron activity. Error bars indicate s.e.m. from three independent cultures. P-values are given above bars. Results of paired t-tests measuring the significance of bortezomib-induced stabilization are indicated with thick lines. The result of a t-test for particular contrast is indicated with thin lines; this assesses how significantly different HUL5 deletion-induced stabilization is in ROLD compared to ROLD bud27Δ. (F and G) Spot assay of yeast strains grown under indicated conditions. (TIF) [file pone.0227841.s003.tif]

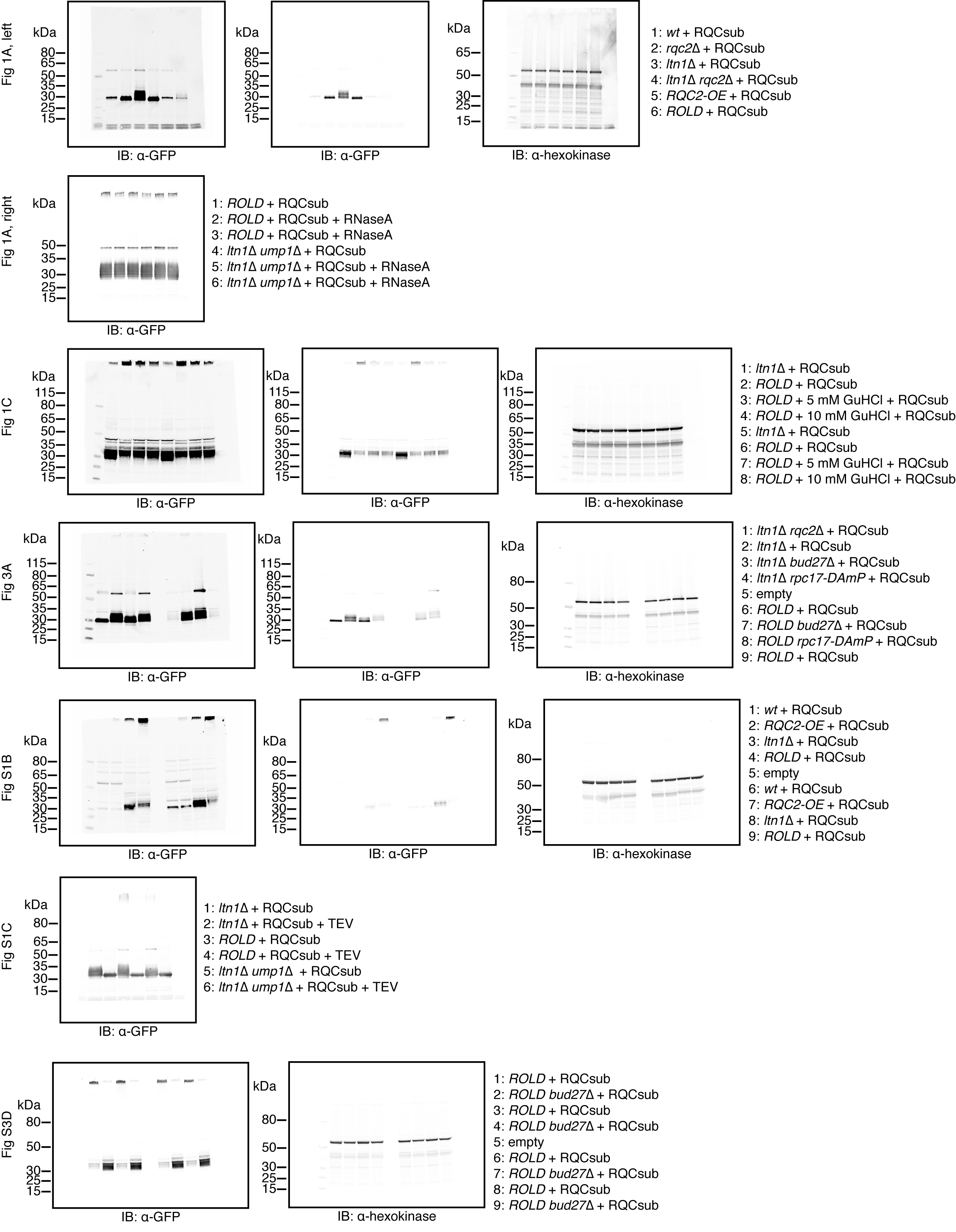

Supplement: S1 Raw Images — (TIF) [file pone.0227841.s007.tif]
