# Supplementary material for: Aggregation of CAT tails blocks their degradation and causes proteotoxicity in S. cerevisiae
Source: PLoS One. 2020 Jan 16;15(1):e0227841. doi: 10.1371/journal.pone.0227841 (PMC6964901; doi:10.1371/journal.pone.0227841)
Supplement: S1 Table — (PDF) [file pone.0227841.s004.pdf]

Supplementary Table 1. Yeast strains used in this study.

Strain Number	Description
yOB199	BY4741
yOB259	BY4741 *ltn1Δ::kanMX*
yOB454	BY4741 *rqc2Δ::kanMX*
yOBj004	BY4741 *ltn1Δ::kanMX rqc2Δ::natMX*
yOB518	BY4741 *pRQC2::HIS3-pTDH3*
yOB551	BY4741 *pRQC2::HIS3-pTDH3 ltn1Δ::natMX*
yOB255	BY4741 *ura3::HSE-EmGFP*
yOB549	BY4741 *ura3::HSE-EmGFP rqc2Δ::kanMX*
yOB550	BY4741 *ura3::HSE-EmGFP ltn1Δ::kanMX*
yOBj245	BY4741 *ura3::HSE-EmGFP rqc2Δ::kanMX ltn1Δ::natMX*
yOB515	BY4741 *ura3::HSE-EmGFP pRQC2::HIS3-pTDH3*
yOBj185	BY4741 *ura3::HSE-EmGFP pRQC2::HIS3-pTDH3 ltn1Δ::natMX*
yOB1118	BY4741 *RQC2-WT::LEU ltn1Δ::hphMX*
yOB2825	BY4741 *RQC2-WT::LEU ltn1Δ::hphMX hul5Δ::natMX*
yOB3812	BY4741 *pRQC2::HIS3-pTDH3 ltn1Δ::natMX hul5Δ::hphMX*
yOBj482	BY4741 *ura3::HSE-EmGFP ump1Δ::natMX*
yOBj538	BY4741 *ura3::HSE-EmGFP ump1Δ::natMX bud27Δ::kanMX*
yOB738	BY4741 *ura3::HSE-EmGFP ump1Δ::natMX rpc17-DAmP::natMX*
yOB736	BY4741 *ura3::HSE-EmGFP hsp104Δ::natMX*
yOB808	BY4741 *ura3::HSE-EmGFP hsp104Δ::natMX bud27Δ::kanMX*
yOB809	BY4741 *ura3::HSE-EmGFP hsp104Δ::natMX rpc17-DAmP::kanMX*
yOBj542	BY4741 *ura3::HSE-EmGFP hsc82Δ::natMX*
yOB818	BY4741 *ura3::HSE-EmGFP hsc82Δ::natMX bud27Δ::kanMX*
yOB722	BY4741 *ura3::HSE-EmGFP hsc82Δ::natMX rpc17-DAmP::kanMX*
yOBj563	BY4741 *ura3::HSE-EmGFP ssa2Δ::natMX*
yOB819	BY4741 *ura3::HSE-EmGFP ssa2Δ::natMX bud27Δ::kanMX*
yOB725	BY4741 *ura3::HSE-EmGFP ssa2Δ::natMX rpc17-DAmP::kanMX*
yOBj536	BY4741 *ura3::HSE-EmGFP bud27Δ::kanMX*
yOB710	BY4741 *ura3::HSE-EmGFP rpc17-DAmP::kanMX*
yOBj536	BY4741 *ura3::HSE-EmGFP bud27Δ::kanMX*
yOBj512	BY4741 bud27Δ::natMX
yOB4036	BY4741 *rpc17-DAmP::kanMX*
yOBj513	BY4741 *ltn1Δ::kanMX bud27Δ::kanMX*
yOB4038	BY4741 *ltn1Δ::kanMX rpc17-DAmP::hphMX*
yOBj495	BY4741 *pRQC2::HIS3-pTDH3 ltn1Δ::natMX bud27Δ::kanMX*
yOBj556	BY4741 *pRQC2::HIS3-pTDH3 ltn1Δ::natMX rpc17-DAmP::kanMX*
yOBj185	BY4741 *ura3::HSE-EmGFP pRQC2::HIS3-pTDH3 ltn1Δ::natMX bud27Δ::kanMX*
yOBj556	BY4741 *ura3::HSE-EmGFP pRQC2::HIS3-pTDH3 ltn1Δ::natMX rpc17-*

	DAmP::kanMX
yOB3812	BY4741 *pRQC2::HIS3-pTDH3 ltn1Δ::natMX hul5Δ::hphMX bud27Δ::kanMX*
OB268	MATα *his3Δ1 leu2Δ0 ura3::4xHSE-EmGFP cyh2 can1Δ::STE2pr-spHIS5 lyp1Δ::STE3pr-LEU2*
OB282	MATα *his3Δ1 leu2Δ0 ura3Δ0 rps0a::URA3-4xHSE-EmGFP cyh2 can1Δ::STE2pr-spHIS5 lyp1Δ::STE3pr-LEU2 ltn1::natMX*
