# Supplementary material for: Aggregation of CAT tails blocks their degradation and causes proteotoxicity in S. cerevisiae
Source: PLoS One. 2020 Jan 16;15(1):e0227841. doi: 10.1371/journal.pone.0227841 (PMC6964901; doi:10.1371/journal.pone.0227841)
Supplement: S2 Table — (PDF) [file pone.0227841.s005.pdf]

Supplementary Table 2. Plasmids used in this study.

Plasmid Name	Description	Selection Marker
RQCsub	CEN/ARS pTDH3-GFP_TEV_R12_RFP	*URA3*
RQCsub (no RFP)	CEN/ARS pTDH3-GFP_TEV_R12_3xHA_3xMYC_3xFLAG	*URA3*
RQCsubLONG	CEN/ARS pTDH3-mKate2-2xT2A-GFP_TEVlonglinker_R12-3xMYC	*URA3*
pRS315	CEN/ARS empty vector	*LEU2*
pJP014	CEN/ARS pRQC2-RQC2WT	*LEU2*
pJP030	CEN/ARS pTDH3-rqc2aaa (D9A, D98A, R99A)	*LEU2*
pJP024	CEN/ARS pRQC2-RQC2WT-mKate2	*LEU2*
pJP055	CEN/ARS pTDH3-RQC2WT-mKate2	*LEU2*
Stalling Reporter	CEN/ARS pTDH3-GFP-2xT2A-TEV_R12-2xT2A-mKate2	*URA3*
Non-stalling Reporter	CEN/ARS pTDH3-GFP-2xT2A-TEV_6xST-2xT2A-mKate2	*URA3*
